# Genome Sequence of Rough and Smooth Variants of Pleomorphic Strain *Lactobacillus farciminis* CNCM-I-3699

**DOI:** 10.1128/genomeA.01059-15

**Published:** 2015-09-17

**Authors:** R. Tareb, M. Bernardeau, J. P. Vernoux

**Affiliations:** a Research Unit EA 4651 Aliments Bioprocédés Toxicologie Environnements (ABTE), IFR146 ICORE, Université de Caen, Basse-Normandie, Esplanade de la paix, Caen, France; b DuPont, Industrial Biosciences, Danisco Animal Nutrition, Marlborough, United Kingdom

## Abstract

The probiotic *Lactobacillus farciminis* CNCM-I-3699 is a pleomorphic strain exhibiting smooth and rough variants. We report their complete genomes consisting of a chromosome of 2, 4 Mb and a plasmid of 6,417 bp. The smooth variant differs by the presence of an additional plasmid of 35,418 bp.

## GENOME ANNOUNCEMENT

*Lactobacillus farciminis* CNCM-I-3699 is a probiotic strain isolated from goat rumen and used now as a probiotic additive in feed (1, 2). The probiotic properties of this strain were demonstrated *in vitro* (3, 4) and *in vivo* (2). Interestingly, *L. farciminis* CNCM-I-3699 is a pleomorphic strain reproducibly giving rise to two phenotypically distinct morphotypes R for rough (*L. farciminis* CNCM-I-3699-R) and S for smooth (*L. farciminis* CNCM-I-3699-S) (5). Significant differences in capsular polysaccharide production were observed: the smooth phenotype corresponds to circular colony with slimy filamentous aspect “ropy”; while the rough morphotype gives irregular, flat, and nonropy colonies. Here we present the complete genome sequence of R and S variants. The only other *L. farciminis* genome available to date is the draft genome of strain KTCC 3126 (6).

Genomic DNA from both variants was first sequenced using the Illumina GAIIx platform (Baseclear, Netherlands) with a 100 bp paired-end library and a total coverage rate that exceeds 100-fold. Assembly of Hiseq reads was performed using CLC Genomics Workbench 5.0 (Genostar, Montbonnot, France). To complement both draft genomes, a 454 pyrosequencing run was performed, targeting 8× coverage, using a GS-FLX sequencer (Genoscreen, Lille, France) and GS-FLX read assembly was done using 454 Newbler version 2.6. Furthermore, an optical map was generated for the smooth variant (Genoscreen, France) using a NcoI enzyme. To generate an orderly genome for both variants, assembled GS-FLX and Hiseq contigs were aligned with the optical map generated from CNCM-I-3699-S.

Both smooth and rough variants exhibit an identical genome, which contains a circular chromosome of 2, 4 Mb (2,423,489 bp and 2,423,335 bp, respectively; G+C 35.7%) and one plasmid of 6,417 bp. The smooth variant differs by the presence of an additional plasmid of 35,418 bp.

Automatic annotation was performed by the RAST annotation server to predict coding DNA sequences (CDS) and their putative functions (7). Gene ontology and Pfm were assigned by searching all predicted proteins against the UFO web server (http://ufo.gobics.de) (8). The predicted proteins were searched against the KEGG database using KAAS (KEGG Automatic Annotation Server http://www.genome.jp/kegg/kaas/) (9). The Prophinder (http://aclame.ulb.ac.be/Tools/Prophinder/) (10) and IS finder (http://www-is.biotoul.fr) (11) were used to detect atypical genome regions corresponding to a prophage, putative horizontal gene transfer, insertion sequence (IS). Clustered regularly interspaced short palindromic repeat (CRISPR) elements already reported (5) were confirmed by the CRISPR Finder web software (12). The specific plasmid of the smooth variant comprises a capsular polysaccharide locus that was flanked by phage sequences. In addition, it includes a sugar transporter and genes related to growth in anaerobic conditions, potentially explaining the predominance of the S variant in anaerobic conditions (5). A more comprehensive report, comparing the complete genomes of the two variants in relation to probiotic properties, genome plasticity, and probiotic lifestyle, will be the subject of a future publication.

### Nucleotide sequence accession numbers.

The complete genome sequences of the chromosomes and plasmids of S and R variants have been deposited in DDBJ/ENA/GenBank under the accession numbers CP011952, CP011953, CP011954, CP012177, and CP012178.

## References

[1] European Union 2008 Commission Regulation (EC) No 1290/2008 of 18 December 2008 concerning the authorization of a preparation of *Lactobacillus rhamnosus* (CNCM-I-3698) and *Lactobacillus farciminis* (CNCM-I-3699) (Sorbiflore) as a feed additive. Official J European Union. http://eur-lex.europa.eu/legal-content/EN/TXT/?uri=uriserv:OJ.L_.2008.340.01.0020.01.ENG.

[2] Owusu-Asiedu A, Bernardeau M, Nyachoti M, Lizardo R, Brufau J, Baidoo S, Dusel G, Simmins H 2012 Influence of supplementing diets with dead forms of beneficial bacterial strains on growth performance of post-weaned- pigs. Oral Presentation, International Congress of Probiotic, Košice, Slovakia.

[3] Bernardeau M, Gueguen M, Smith DGE, Corona-Barrera E, Vernoux JP 2009 *In vitro* antagonistic activities of *Lactobacillus* spp. against *Brachyspira hyodysenteriae* and *Brachyspira pilosicoli*. Vet Microbiol 138:184–190. doi:10.1016/j.vetmic.2009.03.020. 19356863

[4] Tareb R, Bernardeau M, Gueguen M, Vernoux JP 2013 *In vitro* characterization of aggregation and adhesion properties of viable and heat-killed forms of two probiotic *Lactobacillus* strains and interaction with foodborne zoonotic bacteria, especially *Campylobacter jejuni*. J Med Microbiol 62:637–649. doi:10.1099/jmm.0.049965-0. 23329323

[5] Tareb R, Bernardeau M, Horvath P, Vernoux JP 2015 Rough or smooth morphotypes isolated from *Lactobacillus farciminis* CNCM-I-3699 are two closely related variants. Int J Food Microbiol 193:82–90. 25462927 10.1016/j.ijfoodmicro.2014.08.036

[6] Nam SH, Choi SH, Kang A, Kim DW, Kim RN, Kim A, Kim DS, Park H-S 2011 Genome sequence of Lactobacillus farciminis KCTC 3681. J Bacteriol 193:1790–1791. doi:10.1128/JB.00003-11. 21257766 PMC3067648

[7] Aziz RK, Bartels D, Best AA, DeJongh M, Disz T, Edwards RA, Formsma K, Gerdes S, Glass EM, Kubal M, Meyer F, Olsen GJ, Olson R, Osterman AL, Overbeek RA, McNeil LK, Paarmann D, Paczian T, Parrello B, Pusch GD, Reich C, Stevens R, Vassieva O, Vonstein V, Wilke A, Zagnitko O 2008 The RAST server: Rapid Annotations using Subsystems Technology. BMC Genomics 9:75. doi:10.1186/1471-2164-9-75. 18261238 PMC2265698

[8] Meinicke P 2009 Ufo: a web server for ultra-fast functional profiling of whole genome protein sequences. BMC Genomics 10:409. doi:10.1186/1471-2164-10-409. 19725959 PMC2744726

[9] Moriya Y, Itoh M, Okuda S, Yoshizawa AC, Kanehisa M 2007 KAAS: an automatic genome annotation and pathway reconstruction server. Nucleic Acids Res 35:W182–W185. doi:10.1093/nar/gkm321. 17526522 PMC1933193

[10] Lima-Mendez G, Van Helden J, Toussaint A, Leplae R 2008 Prophinder: a computational tool for prophage prediction in prokaryotic genomes. Bioinformatics 24:863–865. 18238785 10.1093/bioinformatics/btn043

[11] Siguier P, Perochon J, Lestrade L, Mahillon J, Chandler M 2006 ISfinder: the reference centre for bacterial insertion sequences. Nucleic Acids Res 34:D32–D36. doi:10.1093/nar/gkj014. 16381877 PMC1347377

[12] Grissa I, Vergnaud G, Pourcel C 2007 CRISPRFinder: a web tool to identify clustered regularly interspaced short palindromic repeats. Nucleic Acids Res 35:W52–W57. doi:10.1093/nar/gkm360. 17537822 PMC1933234

